# The interplay of iron, oxidative stress, and α-synuclein in Parkinson’s disease progression

**DOI:** 10.1186/s10020-025-01208-3

**Published:** 2025-04-26

**Authors:** Yan Chen, Xixi Luo, Yukun Yin, Elizabeth Rosalind Thomas, Kezhi Liu, Wenjun Wang, Xiang Li

**Affiliations:** 1 Department of Psychiatry, The Affiliated Zigong Hospital, Zigong Mental Health Center, Zigong Institute of Brain Science, Southwest Medical University, Luzhou, 646000 China; 2 Department of Biochemistry and Molecular Biology, School of Basic Medical Sciences, Southwest Medical University, Luzhou, 646000 China; 3 Department of Dermatology, The Affiliated Hospital, Southwest Medical University, Luzhou, 646000 China; 4 Department of Medical Microbiology, PGIMER, Chandigarh, 160012 India; 5 Health Science Center, Xi’an Jiaotong University, Xi’an, 710061 China

**Keywords:** Parkinson's disease, α-synuclein, Ferroptosis, Iron

## Abstract

The irreversible degeneration of dopamine neurons induced by α-synuclein (α-syn) aggregation in the substantia nigra is the central pathological feature of Parkinson's disease (PD). Neuroimaging and pathological autopsy studies consistently confirm significant iron accumulation in the brain of PD patients, suggesting a critical role for iron in disease progression. Current research has established that iron overload induces ferroptosis in dopaminergic neurons, evidence indicates that the impact of iron on PD pathology extends beyond ferroptosis. Iron also plays a regulatory role in modulating α-syn, affecting its aggregation, spatial conformation, post-translational modifications, and mRNA stability. Iron-induced α-syn aggregation can contribute to dopaminergic neurodegeneration through additional mechanisms, potentially creating a feedback loop in which α-syn further enhances iron accumulation, thus perpetuating a vicious cycle of neurotoxicity. Given α-syn’s intrinsically disordered structure, targeting iron metabolism presents a promising therapeutic strategy for PD. Therefore, the development of iron chelators, alone or in combination with other therapeutic drugs, may offer a beneficial approach to alleviating PD symptoms and slowing disease progression.

## Introduction

Parkinson's disease (PD) is a progressive neurodegenerative disorder primarily impacting motor function (Aubignat and Tir 2024). With an aging global population, the prevalence of PD continues to rise, making it an important public health issue worldwide. According to the Global Burden of Disease Study 2019, more than 8.5 million people globally are affected by PD (Xu et al. 2024). First described by Dr. James Parkinson in 1817, PD has remained challenging to treat due to its complex pathology and the difficulty in diagnosing it during its earliest stages. The main pathological feature of PD is the degeneration of neurons in the substantial nigra (SN), largely driven by the accumulation of abnormal α-synuclein (α-syn) (Calabresi et al. 2023). The protein buildup ultimately leads to cellular damage and neuronal death. Because the early clinical symptoms of PD are atypical and often go unrecognised, optimal treatment windows are frequently missed.

**Fig. 1:**
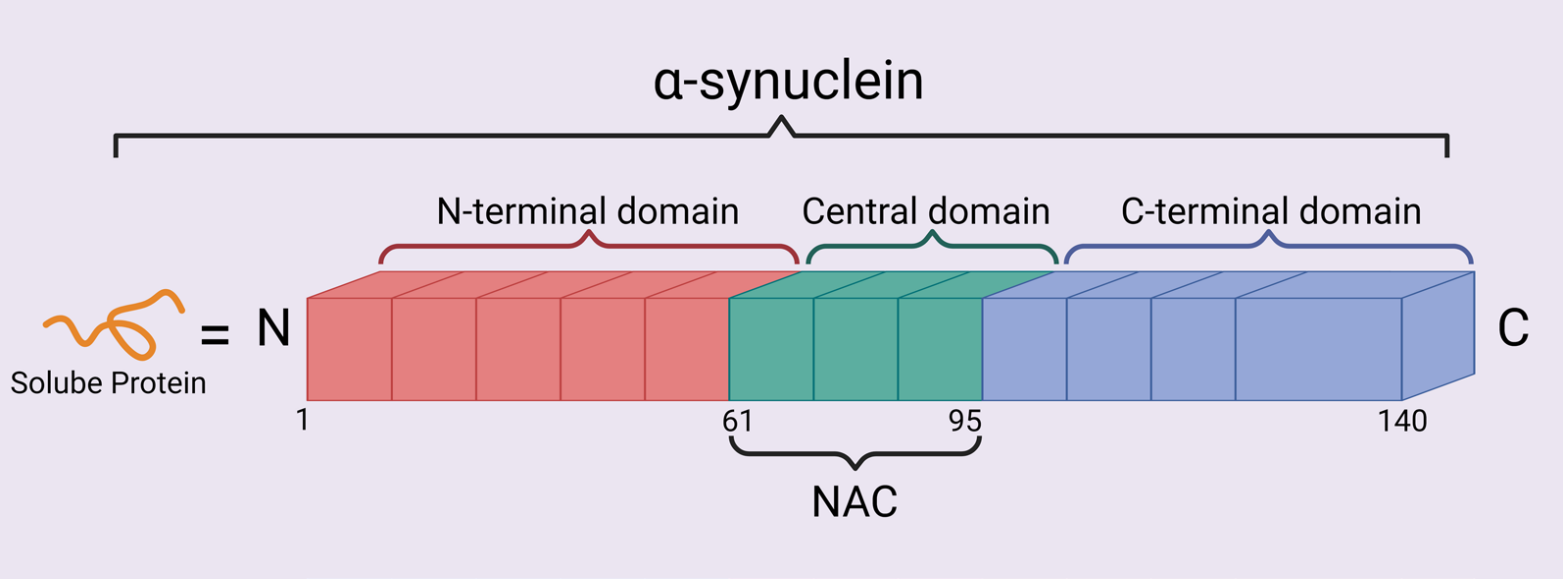
Schematic representation of α-syn structure, illustrating its functional domains: the N-terminal domain (residues 1–60), the central NAC region (residues 61–95), and the C-terminal domain (residues 96–140)

Under normal physiological conditions, α-syn is a small, soluble protein with no neurotoxic effect, predominantly found in neuronal synapses, where it is involved in neurotransmitter release and vesicular recycling (Gao et al. 2023). However, in PD, misfolded α-syn aggregates into prefibrillar structures, eventually forming fibrillar α-syn that constitutes the core of Lewy bodies, a hallmark of PD pathology, which ultimately results in neuronal death (Picca et al. 2021; Leitao et al. 2021; Li et al. 2024). α-syn is mainly expressed in presynaptic nerve terminals and is strongly associated with PD. Structurally, α-syn consists of 140 amino acids organised into three domains: the N-terminal (residues 1–60), the central non-amyloid component (NAC, residues 61–95), and the C-terminal (residues 96–140) (Sarchione et al. 2021) (Fig. 1). Various post-translational modifications (PTMs) such as—phosphorylation, ubiquitination, acetylation and nitration—alter α-syn’s biochemical properties (Yoo et al. 2022), affecting its tendency to aggregate, which is pivotal for the diagnosis and treatment of PD (Guhathakurta et al. 2017). In addition, changes in the spatial conformation of α-syn cause α-syn aggregation into Lewy bodies and is associated with neurotoxicity (Calabresi et al. 2023). Factors like oxidative stress and genetic mutations also influence the aggregation of α-syn, increasing its neurotoxicity in PD.

Epidemiological studies have found that long-term exposure to certain metal ions—such as iron, copper, manganese, and aluminium—is a risk factor for PD (Raj et al. 2021; Bisaglia and Bubacco 2020). Among these, iron is especially implicated as it reveals a higher iron deposition in the dopaminergic neurons of the SN in PD patients compared to healthy individuals (Mochizuki et al. 2020). Becker et al. reported that increased iron levels are detected early in PD, preceding the onset of clinical symptoms, as demonstrated by transcranial color-coded sonography (Becker et al. 1995). People with increased iron in the substantia nigra had a 17 times higher risk of developing PD than those with normal iron levels (Berg et al. 2011). In neurons, iron overload, generally in the form of Fe^2+^, can trigger the Fenton reaction, resulting in lipid peroxidation and neuronal ferroptosis (Jacquemyn et al. 2024), a form of iron dependent cell death. Iron overload is the result of synergistic action of multiple pathways, with the central mechanism being excessive iron uptake mediated by transferrin receptor (TfR) and divalent metal transporter 1 (DMT1) (Lane et al. 2015). While much research has largely focused on inhibiting iron-mediated ferroptosis to ameliorate PD, recent findings suggest that Fe^2+^ and Fe^3+^ may also modulate α-syn through ferroptosis—independent mechanisms (Moons et al. 2020). The interaction between Fe^2+^/Fe^3+^ and α-syn significantly affects the stability and aggregation of α-syn (Wang et al. 2024), influencing disease progression. More importantly, neuronal ferroptosis occurs before neuronal apoptosis in the development of PD (Zhang et al. 2020), which suggests that early improvement of iron accumulation is the key to treating PD.

Elucidating the mechanisms by which iron contribute to α-syn aggregation in a ferroptosis-independent manner is critical. This review explores the role of Fe^2+^ and Fe^3+^ in regulating α-syn aggregation, conformation and modification through alternative pathways unrelated to ferroptosis. By understanding these pathways we aim to provide new insights for early diagnosis and potential therapeutic strategies for PD.

### The role of iron in regulating α-syn aggregation

The aggregation of α-syn in neurons is an important pathological feature of PD. Inhibition of α-syn aggregation is considered a promising therapeutic approach for PD management (Hideshima et al. 2022). Under normal physiological conditions, cellular mechanisms such as autophagy, the ubiquitin proteasome pathway, and the unfolded protein response help degrade misfolded proteins. However, excess Fe^2+^ and Fe^3+^ disrupts these protective pathways, promoting aggregation of α-syn and ultimately leading to neuronal death (Chen et al. 2019; Le 2014; Tang and Kroemer 2020). Beyond ferroptosis, exogenous Fe^2+^ and Fe^3+^ can independently promote aggregation of α-syn and increase its toxicity in a time- and concentration-dependent manner (Li et al. 2010).

Studies have shown that iron overload exacerbates α-syn pathology. For instance, in *Drosophila* models expressing the α-syn A53 T mutant, iron (ferric ammonium citrate, FAC) accumulation exacerbates motor dysfunction (Zhu et al. 2016), underscoring iron’s role in the α-syn aggregation and neurotoxicity. Similarly, in BE-M17 cells overexpressing α-syn, Fe^2+^ enhances its aggregation, leading to the formation of Lewy bodies (Ostrerova-Golts et al. 2000). Transmission electron microscopy (TEM) studies have revealed that, unlike copper (Cu^2+^), which induces amorphous aggregates in mutant α-syn with no fibril formation, Fe^3+^ triggers the formation of short, thick fibrils (Bharathi and Rao 2007). This suggests that aggregation of α-syn is metal-specific and iron (Fe^2+^ and Fe^3+^)-induced aggregation being particularly toxic compared to copper—induced aggregation (Li et al. 2022). Confocal single-molecule fluorescence analysis shows that Fe^3+^ induces aggregation of α-syn and induces the formation of large number of oligomers, which serve as seeds for amyloid fibril formation (Kostka et al. 2008). Notably, this process appears to be independent of the Fenton reaction and depends only on the direct interaction between Fe^3+^ and α-syn (Levin et al. 2011). Even Fe^3+^ at physiological concentrations still promotes the formation of α-syn oligomers (Kostka et al. 2008). Additionally, Fe^2+^ has been found to promote the aggregation of α-syn, while Fe^3+^ exhibits a concentration-dependent effect: at high concentrations, Fe^3+^ inhibits α-syn fibrillation, but at lower concentrations, it promotes α-syn fibrillation (Zhao et al. 2023). Cryo-electron microscopy (cryo-EM) study shows that low concentrations of Fe^3+^ bind to the negatively charged clefts of α-syn fibrils, promoting further aggregation. In contrast, TEM imaging reveals that high concentrations of Fe^3+^ leads to formation of spherical α-syn oligomers, which prevents further assembly of amyloid fibrils (Zhao et al. 2023). Thioflavin (ThT) kinetic trajectory and circular dichroism studies show that the binding of Fe3+ to α-syn increases the rate of fiber formation and leads to more secondary structures (β-sheets) (Uversky et al. 2001). These findings suggest that Fe^3+^ plays a more direct role in promoting the aggregation of α-syn (Uversky et al. 2001; Peng et al. 2010), whereas Fe^2+^ primarily contributes to α-syn aggregation by generating high levels of reactive oxygen species (ROS) through the Fenton reaction.

### The role of iron in promoting α-syn aggregation by regulating its conformation

Altered α-syn conformation in neurons is a key factor in α-syn aggregation during PD. The N-terminal domain of α-syn, which is positively charged, interacts with the phospholipid bilayer to form α-helical structures (Musteikyte et al. 2021). The NAC domain regulates protein fibrillation (Gallardo et al. 2020), while negatively charged C-terminal domain also contributes to α-syn aggregation (Emamzadeh 2016; Zhang et al. 2022). The C-terminal domain of α-syn is rich in acidic amino acids, such as glutamate (Glu) and aspartate (Asp), giving it a negative charge under physiological conditions (Emamzadeh 2016). Metal ions interacting with the C-terminal of α-syn tend to expose the NAC region, accelerating fibril formation (Atarod et al. 2022). Conformational shifts in α-syn are crucial for synaptic transmission: while physiological shifts facilitate neurotransmitter release and vesicle recycling, pathological aggregation leads to synaptic dysfunction and promotes neuronal death. The extent and nature of α-syn’s conformational changes directly correlate with PD progression and symptom severity (So and Watts 2023; Tofaris 2022).

**Fig. 2:**
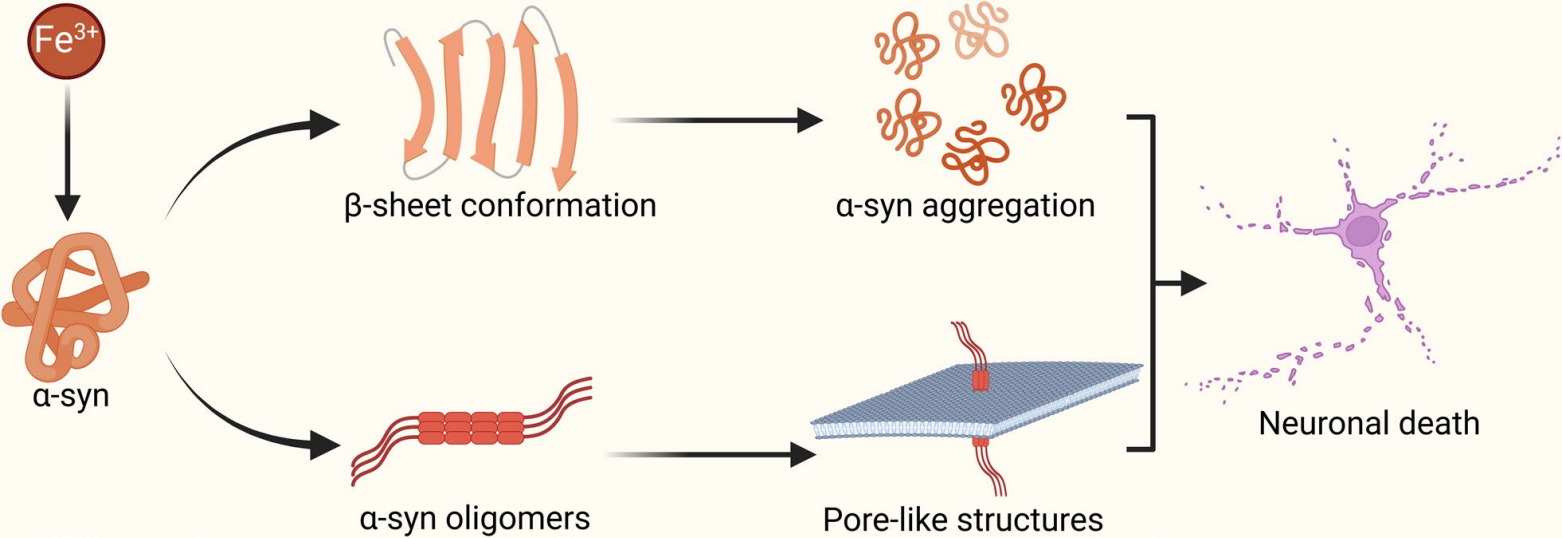
Fe^3+^ alters the spatial conformation of α-syn, converting it into a β-sheet structure, which enhances its aggregation. Fe^3+^ promotes the formation of α-syn oligomers and pore-like structures in the neuronal membranes, leading to altered membrane permeability and neuronal death

Research on iron’s role in promoting α-syn aggregation remains limited, but emerging evidence highlights key interactions. The nuclear magnetic resonance (NMR) analysis demonstrates that Fe^2+^ can bind to the C-terminal region of α-syn (Binolfi et al. 2006), altering the spatial conformation of α-syn and promoting its aggregation (Abeyawardhane et al. 2018). Similarly, Fe^3+^ induces a shift in α-syn structure from an α-helical to a β-sheet conformation, enhancing its propensity to aggregate (Kaindlstorfer et al. 2018) (Fig. 2). Cryo-electron microscopy studies show that Fe^3+^ binds to the negative cleft of α-syn fibrils, stabilizing the fibrillar structure and promoting further aggregation (Zhao et al. 2023). Beyond fibril formation, Fe^3+^ promotes the formation of α-syn oligomers, which can create pore-like structures in planar lipid bilayers (Kostka et al. 2008), altering neuronal membrane permeability and triggering neuronal death (Tsigelny et al. 2012) (Fig. 2). Both Fe^3+^ and Fe^2+^ binds to α-syn, inducing conformational changes that accelerate its aggregation (Moons et al. 2020; Peng et al. 2010). These findings suggest that Fe^2+^ and Fe^3+^ stabilises α-syn fibrils by binding to negatively charged residues via electrostatic interactions, promoting toxic aggregation and contributing to dopamine neuron degeneration in PD.

### The role of iron in promoting α-syn aggregation by inhibiting autophagy

**Fig. 3:**
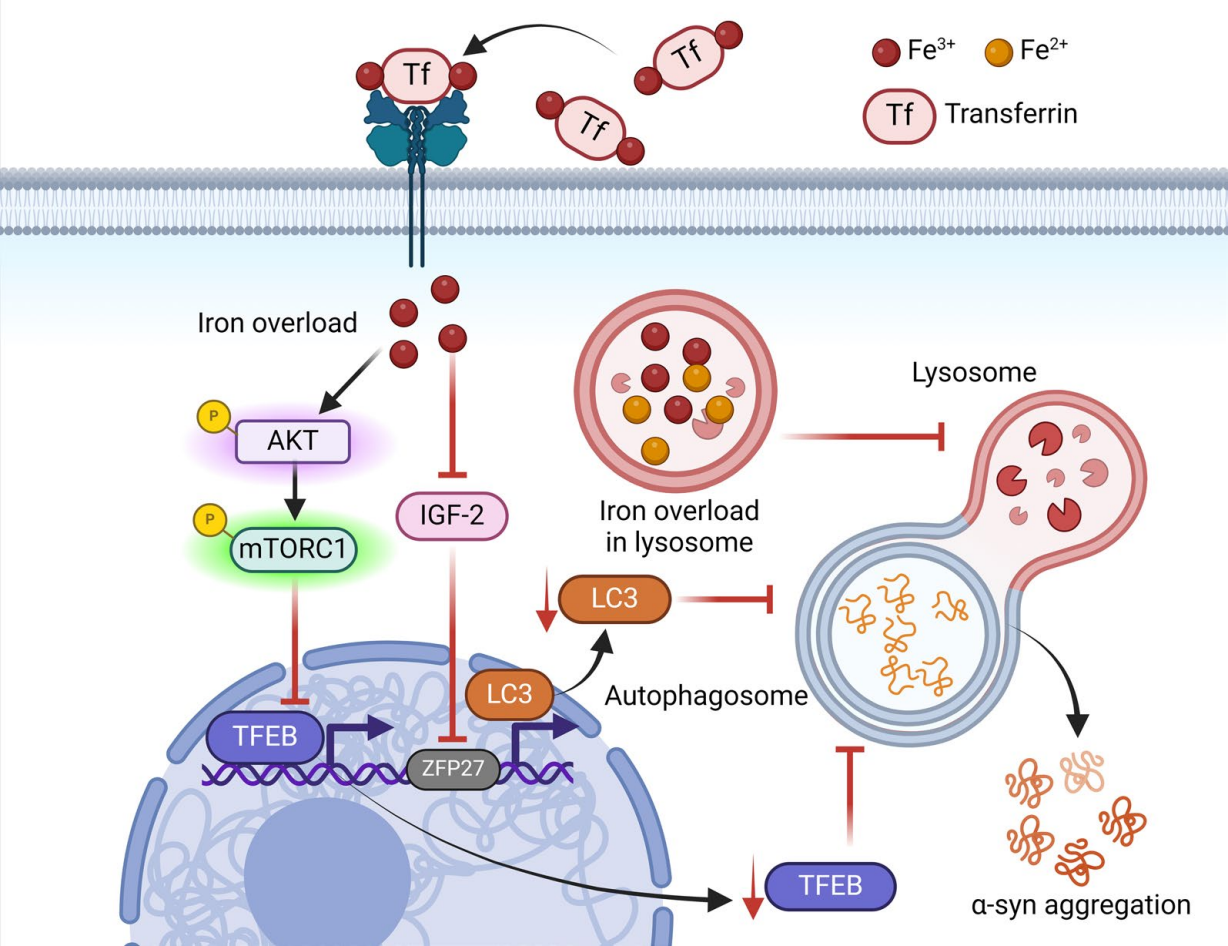
Iron overload activates the AKT/mTORC1 signalling pathway and inhibits TFEB expression, thereby inhibiting autophagy-mediated degradation of misfolded α-syn and leading to neuronal death. Iron overload suppresses IGF2 and ZFP27, which decreased LC3 expression and further impairs autophagy. Iron overload in lysosomes impairs autophagy

Dysfunction in autophagy is a key factor contributing to α-syn accumulation and the progression of PD. Previous studies have reported that iron overload can inhibit autophagy, thereby promoting α-syn accumulation in neurons (Wan et al. 2017; Wu et al. 2017). Specifically, Fe^2+^ overload activates the protein kinase B (AKT)/mechanistic target of rapamycin complex 1 (mTORC1) pathway, while also inhibiting the expression of transcription factor EB (TFEB), a master transcriptional regulator of autophagosome-lysosome fusion. This inhibition exacerbates α-syn aggregation and propagation (Xiao et al. 2018). Additionally, Fe^3+^ deposition suppresses insulin-like growth factor 2 (IGF2) and the transcription factor zinc finger protein 27 (ZFP27), which decreased LC3 expression and further impairs autophagy (Wang et al. 2023) (Fig. 3).

Furthermore, both Fe^2+^ and Fe^3+^ overload lead to iron accumulation within lysosomes, impairing their function and contributing to defective autophagy (Fernandez et al. 2016) (Fig. 3). These results illustrate that iron accumulation disrupts autophagic processes, preventing the clearance of α-syn and ultimately resulting in neuronal loss.

### The role of iron in promoting α-syn aggregation by enhancing α-syn mRNA expression

Iron promotes α-syn aggregation by increasing its mRNA translation. Iron binds to iron regulatory protein (IRP), causing its dissociation from the iron response element (IRE), which enhances the translation of transcripts associated with α-syn (Zhou and Tan 2017). In SK-N-SH cells, IRP knockdown has been shown to upregulate α-syn mRNA expression, resulting in increased α-syn aggregation (Friedlich et al. 2007). Furthermore, the use of iron chelators reduces α-syn mRNA expression in HEK293 cells (Li et al. 2011).

Administration of iron chelators reduce the mRNA expression of α-syn in HEK293 cells (Febbraro et al. 2012). Research by Tong et al. identified Synucleozid-2.0, a small molecule that targets the IRE region of α-syn mRNA, promoting its degradation and reducing α-syn levels (Tong and Zhang 2024). These findings suggest that iron overload facilitates IRP dissociation from α-syn mRNA, enhancing α-syn translation and promoting its aggregation, which worsens PD pathology.

**Fig. 4:**
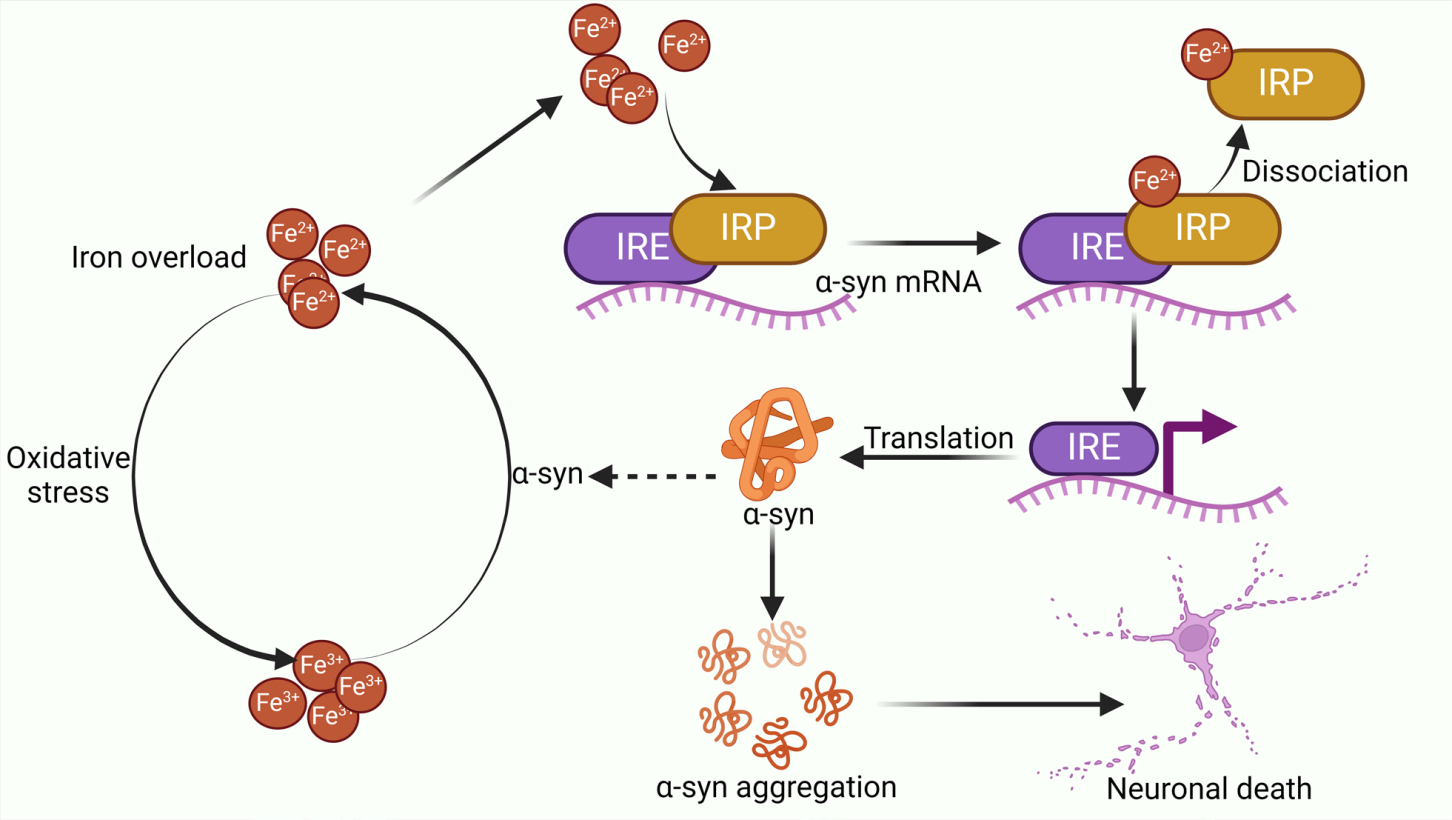
Iron overload binds to the iron regulatory proteins (IRP), causing their release from the iron-responsive element (IRE) on α-syn mRNA, which enhances α-syn translation and contributes to increased protein formation and aggregation. α-syn functions as a ferrireductase, reducing Fe^3+^ to Fe^2+^. Fe^2+^ catalyse H_2_O_2_ to produce Fe^3+^ via the Fenton reaction

Additionally, oxidative stress in PD generates reactive oxygen species (ROS), which oxidise Fe^2+^ to Fe^3+^, further promoting α-syn aggregation (Levin et al. 2011). It's worth noting that α-syn functions as a ferrireductase to reduce Fe^3+^ to Fe^2+^ (Sian-Hulsmann and Riederer 2020). This forms a self-perpetuating cycle of iron-induced α-syn aggregation and neuronal damage, exacerbating PD progression (Fig. 4).

### The role of iron in regulating α-syn propagation

**Fig. 5:**
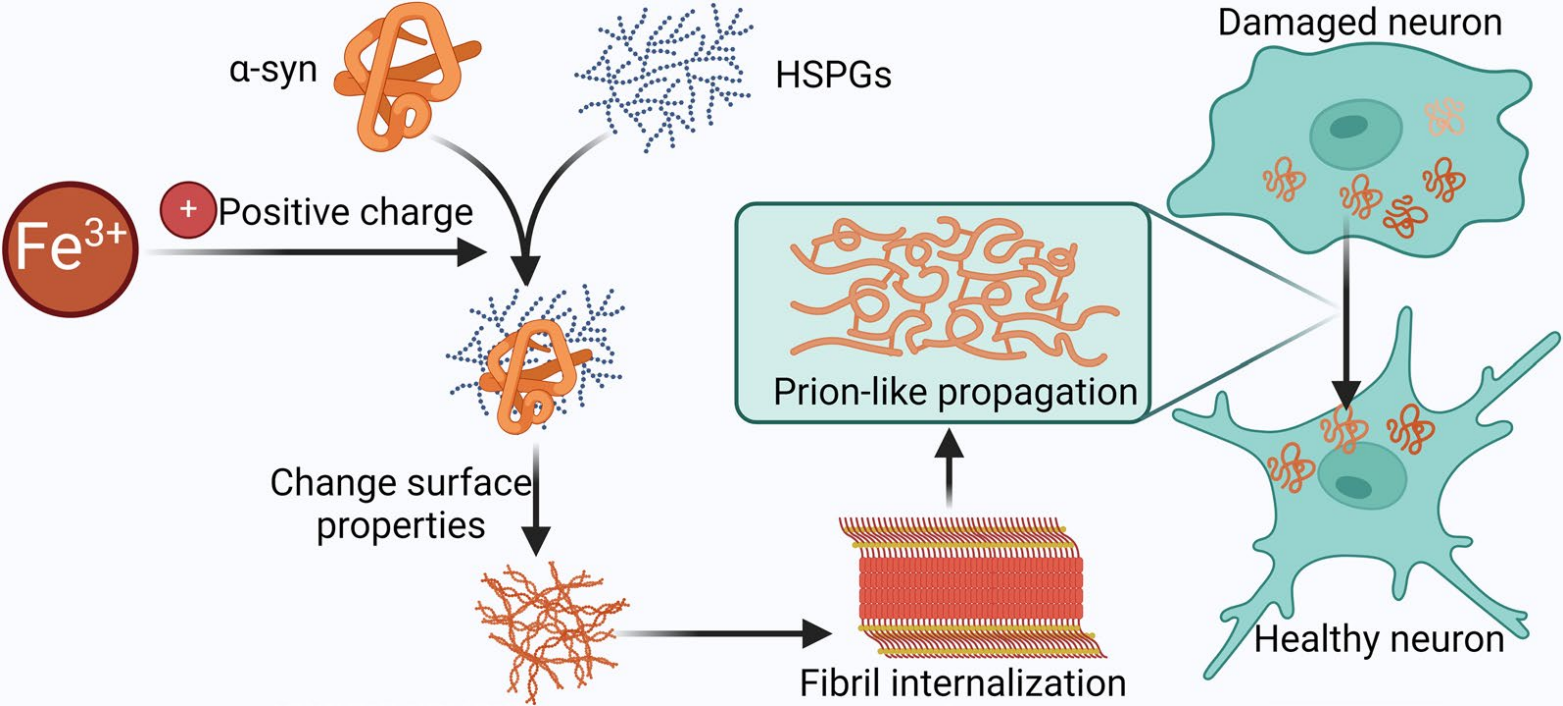
Iron enhances the binding of α-syn to heparin sulfate proteoglycans (HSPGs) through its positive charge, altering the surface properties of α-syn. This promotes fibril internalisation and accelerates the prion-like propagation of α-syn

Research indicates that under pathological conditions, α-syn can be released from the cytoplasm into the extracellular matrix, where it is taken up by neighbouring neurons. This process is especially prevalent for “seed-toxic” oligomers of α-syn, which are more likely to be internalised by recipient neurons (Danzer et al. 2012). Studies have shown that Fe^3+^ promotes the transcellular propagation of α-syn in a concentration-dependent manner (Li et al. 2020). In addition, Fe^3+^ enhances the binding of α-syn to heparan sulfate proteoglycans (HSPGs) through its positive charge, altering the surface properties of α-syn and promoting fibril internalisation. This accelerates the prion-like propagation of α-syn (Li et al. 2020) (Fig. 5).

Although many studies link iron accumulation to increased α-syn levels, the direct mechanisms by which iron promotes α-syn propagation remains unmapped. Nonetheless, these findings suggest that iron plays a critical role in facilitating the spread of α-syn aggregates between cells, contributing to the progression of PD.

### The role of iron in promoting α-synphosphorylation, exacerbating its aggregation

Post-translational modifications of α-syn is essential for its pathological aggregation in PD (Zhang et al. 2021). Among these, phosphorylation of α-syn, particularly at serine 129, is particularly significant. While only a small proportion of α-syn is phosphorylated in healthy brains, over 90% of the abnormally aggregated α-syn in PD patients is phosphorylated at serine 129 (Xu et al. 2015; Kawahata et al. 2022). Although phosphorylation of α-syn has been shown to induce cytotoxicity and promotes the formation of Lewy bodies, its exact role in PD pathology remains ambiguous (Ramalingam et al. 2024; Kontaxi and Edwards 2023).

**Fig. 6:**
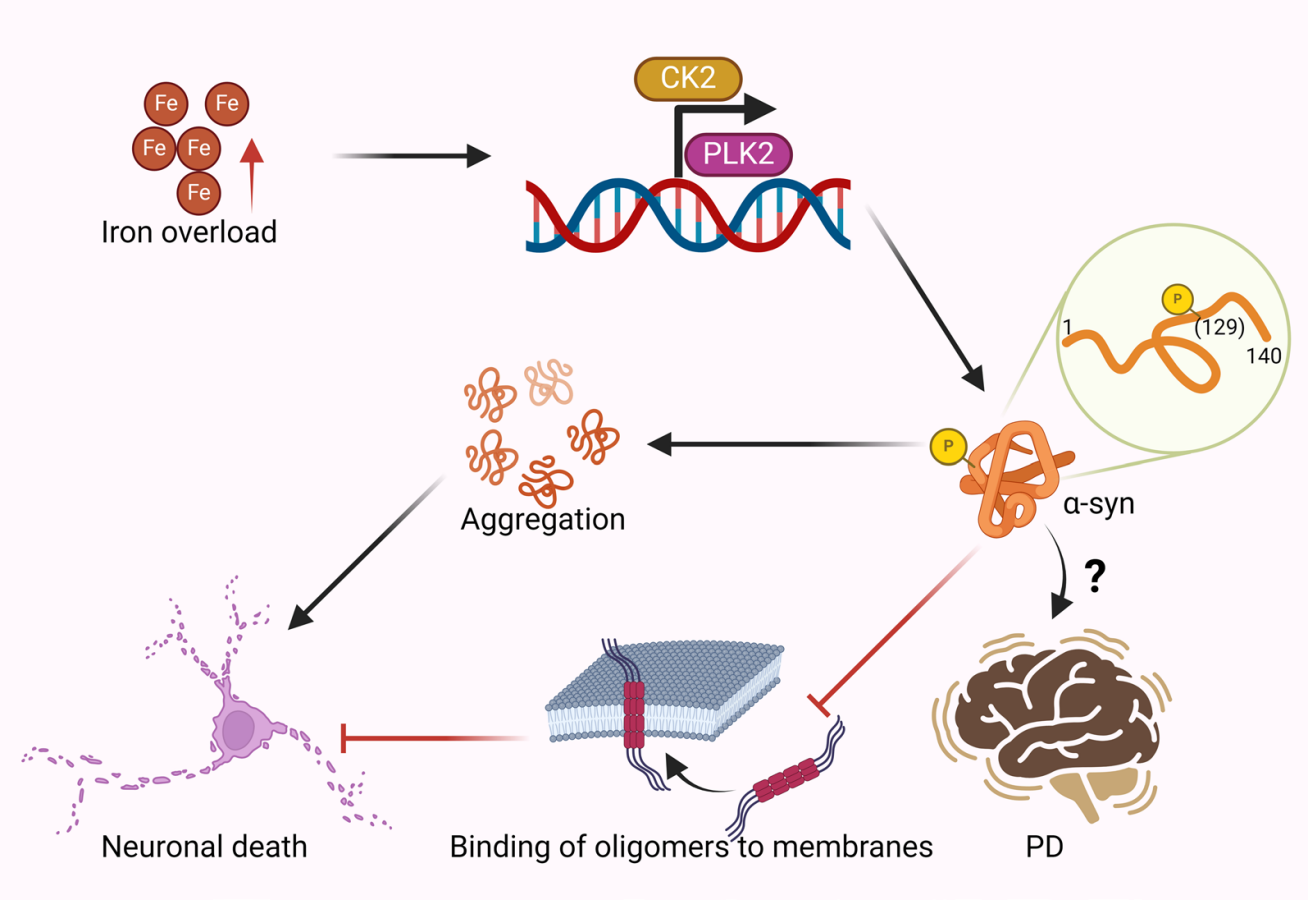
Iron overload induces phosphorylation of α-syn at serine 129, accompanied by elevated expression of CK2 and PLK2, which regulates α-syn phosphorylation at Ser129. Phosphorylation of α-syn at serine 129 prevents α-syn oligomers from binding to the cell membranes, potentially protecting the cell membrane from neurotoxic effects. The precise role of serine 129 phosphorylation in α-syn’s function and toxicity remains unclear

Iron overload induces phosphorylation of α-syn at serine 129, correlating with increased expression of casein kinase 2 (CK2) and polo-like kinase 2 (PLK2), both of which regulate α-syn phosphorylation (Wang et al. 2019; Perfeito et al. 2014). Administration of antioxidants, such as *N*-acetyl-L-cysteine, protects neurons from the oxidative stress associated with iron overload, suggesting that ROS generated by iron may induce phosphorylation of α-syn thereby exacerbating its aggregation (Wang et al. 2019). The functional implication of α-syn phosphorylation at serine 129 is still being explored. Some studies indicate that this modification prevents α-syn oligomers from binding to the cell membranes, potentially protecting the cell membrane from neurotoxic effects (Sato et al. 2013). Others propose that phosphorylation of α-syn at serine 129 occurs during the initial stages of α-syn aggregation, which suppresses further aggregation (Ghanem et al. 2022). Given that iron accumulation occurs early in PD, we suggest that early iron-induced phosphorylation of α-syn is protective. However, as PD progresses, excessive iron accumulation exacerbates phosphorylation and induces α-syn aggregation. Therefore, the impact of serine 129 phosphorylation on PD pathology needs to be further investigated (Fig. 6). Furthermore, while iron’s role in α-syn phosphorylation is well documented, its effect on other post translational modifications, such as acylation and nitration remains unclear. Further research is needed to clarify their contributions to PD progression.

### The role of iron in promoting α-syn aggregation by regulating ROS

Fe^2+^ can effectively catalyse H_2_O_2_ to produce Fe^3+^ and ROS via the Fenton reaction, contributing to oxidative stress (Yan et al. 2022). Interestingly, α-syn itself functions as a ferrireductase, reducing Fe^3+^ to Fe^2+^ (Jansen van Rensburg et al. 2021). This continuous cycle, combined with elevated expression of α-syn, leads to the accumulation of Fe^2+^, resulting in increased ROS production and neuronal toxicity. Davies et al. demonstrated that α-syn overexpression significantly increases ferrireductase activity, increasing the level of Fe^2+^ in SH-SY5Y cells lysates (Davies et al. 2011). Studies further show that the administration of metal chelators such as deferoxamine (DFO) reduces α-syn oligomer-induced ROS accumulation, mitigating oxidative damage and reducing the iron level (Shen et al. 2024; Deas et al. 2016).

While iron accumulation alone is not considered the primary cause of PD, early-life exposure to excess iron increases PD risk later in life (Hare et al. 2015; Zecca et al. 2004). Impaired iron metabolism is particularly detrimental to dopaminergic neurons (Hare and Double 2016), and increased iron levels in the substantia nigra region of the midbrain have been suggested as a potential early marker for PD (Martin et al. 2008). Abnormal aggregation of α-syn causes ROS accumulation, resulting in dopamine neuron death and contributing to PD progression. However, whether ROS accumulation directly causes α-syn aggregation in the early stages of PD is still unclear.

In animal models, long-term administration of MPTP, a neurotoxin used to mimic PD, causes accumulation of α-syn in the substantia nigra region of mice (Zhang et al. 2017). MPTP is metabolised to MPP^+^ by monoamine oxidase B, which inhibits mitochondrial complex I, leading to ROS accumulation and PD-like symptoms. In patients with asymptomatic PD, reduced glutathione levels in the substantia nigra mirror those in advanced PD, suggesting that oxidative stress may precede α-syn aggregation (Jenner et al. 1992).

Early accumulation of ROS may be an important cause of α-syn aggregation, and iron buildup could serve as an early marker of PD by inducing oxidative stress that promotes α-syn aggregation. We propose that ROS buildup plays a central role in the progression of PD by driving α-syn aggregation and exacerbating neuronal damage.

### The role of iron in promoting dopaminergic neuronal death through apoptosis and necroptosis

Iron is essential for various physiological processes, including oxygen transport and energy metabolism. Excess iron leads to oxidative stress, high levels of ROS, and dopaminergic neuronal apoptosis. Intracerebroventricular injection of iron citrate exacerbated MPTP-induced Parkinsonism by promoting apoptosis via the Bcl-2/Bax and activated caspase-3 pathway (You et al. 2015). Ci et al. reported that knockdown of the iron regulatory protein 2 (IRP2) gene induces iron accumulation in the SN region, exacerbating neuronal apoptosis and PD-like symptoms (Ci et al. 2020). We suggest that ferroptosis precedes other iron-mediated neurotoxicity, as ferroptosis inhibitors can mitigate iron-induced apoptosis, whereas apoptosis inhibitors fail to prevent ferroptosis (Zhang et al. 2020). In conclusion, intracellular iron overload causes apoptosis and ferroptosis, and the use of iron chelators such as DFO protects neurons, effectively (Zeng et al. 2021).

In addition, necroptosis contributes to dopaminergic neuronal degeneration in the SN region of PD. Analysis of postmortem brain tissue of PD patients revealed elevated levels of the key necroptotic proteins, including receptor-interacting protein kinase 1 (RIPK1), receptor-interacting protein kinase 3 (RIPK3) and mixed lineage kinase domain-like (MLKL) (Iannielli et al. 2018).. Many studies have confirmed the presence of necroptotic proteins in animal models of PD (Leem et al. 2023, 2024). Necroptosis inhibitors, such as necrostatin-1, protects dopaminergic neurons from 6-hydroxydopamine -induced neurotoxicity (Wu et al. 2015; Onate et al. 2020). Iron overload is known to cause ROS accumulation and induce necroptosis. The use of the ferroptosis inhibitor ferrostatin-1 is more effective for iron overload toxicity induced by iron dextrose compared to necrosis inhibitors and apoptosis inhibitors (Kumfu et al. 2023).

In conclusion, elevated ROS induced by iron overload causes abnormal accumulation of α-syn, ultimately leading to neuronal death. Iron accumulation may precede the clinical onset of PD (Pyatigorskaya et al. 2020; Alushaj et al. 2024) with ferroptosis serving as the main mode of dopaminergic neuronal loss in early PD.

## Discussion

PD is characterised by excessive iron accumulates in the brain, marked by increased levels of iron and iron-binding proteins such as ferritin (Ru et al. 2024). This iron overload, coupled with reduced glutathione levels, indicates disrupted redox homeostasis in patients with PD. Oxidative damage from iron accumulation is a major causes of PD pathogenesis (David et al. 2022), and neuronal ferroptosis due to iron overload is an important hallmark of PD. While current studies largely focuses on improving PD by inhibiting iron-induced toxicity, exploring alternative pathways of iron-related pathology may provide additional therapeutic insights. In particular, aggregation of α-syn and the resulting formation of Lewy bodies remain key pathological feature of PD. We suggest that Fe^3+^ is more effective in promoting the aggregation of α-syn (Uversky et al. 2001; Peng et al. 2010), whereas Fe^2+^ causes α-syn aggregation mainly by generating high levels of ROS through the Fenton reaction. However, Fe^3+^ produced by the Fenton reaction is subsequently reduced to Fe^2+^ by cellular reductase, creating a vicious cycle that further damages dopaminergic neurons.

Dysregulated iron homeostasis promotes oxidative stress and dopaminergic neuronal damage, both of which are central to the pathogenesis of PD (Holbein and Lehmann 2023). Iron accumulation, observed initially in the midbrain and gradually extending to the striatum, correlates with early PD clinical features and diagnosis (Yan et al. 2024). Inhibition or downregulation of transferrin in the central nervous system has been shown to reduce iron levels in the brain, which significantly ameliorates ROS in a 6-OHDA model, allocating oxidative stress (Xue et al. 2020). Elevated expression of divalent metal-ion transporter 1 (DMT1) and IRE in patients with PD significantly contribute to iron accumulation in the SN of patients of PD (Ndayisaba et al. 2019). Furthermore, activation of the mitogen-activated protein kinase (MAPK) pathway by α-syn inhibits ubiquitination of DMT1, promoting DMT1 expression and enhancing iron influx into neurons (Bi et al. 2020). This interplay suggests that aggregation of α-syn initially triggers iron deposition in the brain, exacerbating aggregation of α-syn, creating a detrimental feedback loop that accelerates PD progression. Disrupting this vicious cycle is critical for therapeutic intervention.

Iron overload can activate glial cells, leading to the release neuroinflammatory factors such as IL-1β, TNF-α, and IL-6, affecting neurons (Liu et al. 2022). The release of inflammatory factors promotes the transcription and accumulation of α-syn (Angelova and Brown 2018; Li et al. 2021). In rat neuron-microglia-astroglia cultures, microglia enhance iron-induced selective and progressive toxicity in dopamine neuronsl (Zhang et al. 2014). More importantly, Guo et al. found that iron is specifically deposited in microglia in the SN region, but not in dopaminergic neurons in the PD model of *Macaca fascicularis* induced by α-syn preformed fibrils (Guo et al. 2021). This suggests that iron deposition in microglia is an early response to induce inflammation in dopamine neurons. Additionally, microglia are the most prone to ferroptosis (Ryan et al. 2023). However, it remains unclear whether microglial activation is the primary cause of PD (Oleg and Weiner 2018; Hickman et al. 2018). In a mouse model of lentivirus-mediated accumulation of α-syn in microglia, dopaminergic neuronal damage occurred without endogenous α-syn accumulation (Bido et al. 2021). Since microglia are among the first cells to show iron deposition, addressing iron overload in microglia may be key to preventing PD.

Iron is an important regulator of mitochondrial function, but excess iron can lead to mitochondrial dysfunction. Fe^2+^ catalyses the generation of ROS via the Fenton reaction, leading to lipid peroxidation of mitochondrial membranes, which disrupts mitochondrial membrane integrity and results in mitochondrial dysfunction (Rochette et al. 2022). Iron accumulation interferes with the mitochondrial electron transport chain (ETC), leading to reduced ATP production and impaired energy metabolism, further exacerbating cellular damage (Guo et al. 2022). Lysosomes occupy a central position in iron homeostasis in addition to its role in degradation and recycling. Iron overload also affects lysosomal function and impairs mitophagy, which in turn exacerbates mitochondrial dysfunction. Fe^3+^ stored in lysosomes is reduced to Fe^2+^ in acidic environments, leading to lysosomal oxidative damage, releasing Fe^2+^ and proteases into the cytoplasm and triggering ferroptosis (Rizzollo et al. 2021; Kurz et al. 2011). In addition, lysosomal membranes are rich in polyunsaturated fatty acids, making them susceptible to Fe^2+^-mediated lipid peroxidation, which can directly disrupt membrane integrity. In summary, iron accumulation impairs both mitochondrial and lysosomal function, contributing to α-syn accumulation and inducing PD.

Currently, no approved drug effectively inhibits or degrades α-syn aggregation to treat PD. Due to α-syn’s intrinsically disordered structure, it remains challenging to target it directly with pharmacological agents (Bisi et al. 2021). Improving iron metabolism and limiting iron accumulation are promising alternative approaches to inhibit α-syn aggregation indirectly. Several studies have reported that iron chelators demonstrated positive results in PD models, by reducing α-syn aggregation and limiting iron induced neuronal damage (Jansen van Rensburg et al. 2021). For example, Huang et al. demonstrated that administration of deferoxamine mesylate ameliorated MPTP-induced Parkinsonism in mice (Mannan Thodukayil et al. 2019). A summary of iron chelators used in PD research is provided in Table 1.

**Table 1:** A summary of iron chelators used in PD research

Iron chelator	Model	Mechanism	References
PBT434	6-OHDA, MPTP or A53 T-α-syn -induced PD model in mice	Inhibition of oxidative damage and α-syn accumulation	Schijndel et al. (2017)
VAR10303	6-OHDA or MPTP-induced PD model in mice	Inhibition of apoptosis	Bar-Am et al. (2015)
Deferoxamine	MPTP-induced PD model in mice	Inhibition of ferroptosis	Lei et al. (2024)
Deferiprone	Patients with PD	Inhibition of ferroptosis	Devos et al. (2022)
1,2-HOPO	6-OHDA-induced PD model in SH-SY5Y cells	Inhibition of ferroptosis	Workman et al. (2015)
CN128	PD model in SH-SY5Y cells	Inhibition of ferroptosis	Sun et al. (2020)
HBED and FeTBAP	MPP + -induced PD model in SH-SY5Y cells	Inhibition of apoptosis	Kalivendi et al. (2003)
VK-28	6-OHDA-induced PD model in mice	Inhibition of mitochondrial lipid peroxidation	Youdim et al. (2004)
Deferasirox	6-OHDA-induced PD model in cultured mouse dopaminergic neurons	Inhibition of ferroptosis and promotion of parkin	Ham et al. (2023)
SIH	6-OHDA-induced PD model in PC12 cells	Inhibition of ferroptosis	Haskova et al. (2022)

Iron chelators, particularly when targeting both α-syn aggregation and ferroptosis, demonstrate a promising dual mechanism for PD treatment. Phase II clinical trials of deferiprone (DFP) show potential in improving motor symptoms, however, current evidence remains insufficient to support its routine use in patients with PD (Negida et al. 2024; Martin-Bastida et al. 2017). In addition, a significant limitation of existing iron chelators is their restricted ability to penetrate the blood–brain barrier, which limits their therapeutic efficacy in clinical PD applications. Therefore, developing next-generation iron chelators with enhanced CNS bioavailability or combining chelators with other therapeutic agents represents a crucial research direction for future treatments of PD.

## Conclusion

This review has highlighted the role of iron in regulating α-syn aggregation through alternative pathological pathways.Regulation of iron metabolism not only inhibits α-syn aggregation but also effectively suppresses neuronal ferroptosis, both of which are central to PD pathology. Given the challenging nature of α-syn’s structure, designing drugs that promote its degradation has proven difficult. Targeting iron metabolism may offer a viable alternative and hold promise as an effective treatment for PD. Future research into iron-targeted therapies could pave the way for more successful treatment strategies for PD.

## Data Availability

No datasets were generated or analysed during the current study.

## Abbreviations

PD: Parkinson's disease
α-syn: α-synuclein
SN: Substantial nigra
PTMs: Post-translational modifications
AKT: Protein kinase B
mTORC1: Mechanistic target of rapamycin complex 1
TFEB: Transcription factor EB
IGF2: Insulin-like growth factor 2
ZFP27: Transcription factor zinc finger protein 27
IRP: Iron regulatory protein
IRE: Iron response element
ROS: Reactive oxygen species
HSPGs: Heparan sulfate proteoglycans
CK2: Casein kinase 2
PLK2: Polo-like kinase 2
DMT1: Divalent metal-ion transporter 1
MAPK: Mitogen-activated protein kinase
DFP: Deferiprone
TfR: Transferrin receptor
DMT1: Divalent metal transporter 1
FAC: Ferric ammonium citrate
TEM: Transmission electron microscopy
cryo-EM: Cryo-electron microscopy
ThT: Thioflavin
Glu: Glutamate
Asp: Aspartate
NMR: Nuclear magnetic resonance
DFO: Deferoxamine
RIPK1: Receptor-interacting protein kinase 1
RIPK3: Receptor-interacting protein kinase 3
MLKL: Mixed lineage kinase domain-like
